# Ancient horizontal transfers of retrotransposons between birds and ancestors of human pathogenic nematodes

**DOI:** 10.1038/ncomms11396

**Published:** 2016-04-21

**Authors:** Alexander Suh, Christopher C. Witt, Juliana Menger, Keren R. Sadanandan, Lars Podsiadlowski, Michael Gerth, Anne Weigert, Jimmy A. McGuire, Joann Mudge, Scott V. Edwards, Frank E. Rheindt

**Affiliations:** 1 Department of Evolutionary Biology, Evolutionary Biology Centre (EBC), Uppsala University, SE-752 36 Uppsala, Sweden; 2 Department of Biology and Museum of Southwestern Biology, University of New Mexico, Albuquerque, New Mexico 87131, USA; 3 Department of Conservation Biology, Helmholtz Centre for Environmental Research (UFZ), D-04318 Leipzig, Germany; 4 Molecular Evolution and Systematics of Animals, Institute of Biology, University of Leipzig, D-04103 Leipzig, Germany; 5 Instituto Nacional de Pesquisas da Amazônia (INPA), AM 69067-375 Manaus, Brazil; 6 Department of Biological Sciences, National University of Singapore, Singapore 117543, Singapore; 7 Institute of Evolutionary Biology and Ecology, University of Bonn, D-53121 Bonn, Germany; 8 Max Planck Institute for Evolutionary Anthropology, D-04103 Leipzig, Germany; 9 Museum of Vertebrate Zoology and Department of Integrative Biology, University of California, Berkeley, Berkeley, California 94720, USA; 10 National Center for Genome Resources, Santa Fe, New Mexico 87505, USA; 11 Department of Organismic and Evolutionary Biology, Harvard University, Cambridge, Massachusetts 02138, USA

## Abstract

Parasite host switches may trigger disease emergence, but prehistoric host ranges are often unknowable. Lymphatic filariasis and loiasis are major human diseases caused by the insect-borne filarial nematodes *Brugia*, *Wuchereria* and *Loa*. Here we show that the genomes of these nematodes and seven tropical bird lineages exclusively share a novel retrotransposon, AviRTE, resulting from horizontal transfer (HT). AviRTE subfamilies exhibit 83–99% nucleotide identity between genomes, and their phylogenetic distribution, paleobiogeography and invasion times suggest that HTs involved filarial nematodes. The HTs between bird and nematode genomes took place in two pantropical waves, >25–22 million years ago (Myr ago) involving the *Brugia*/*Wuchereria* lineage and >20–17 Myr ago involving the *Loa* lineage. Contrary to the expectation from the mammal-dominated host range of filarial nematodes, we hypothesize that these major human pathogens may have independently evolved from bird endoparasites that formerly infected the global breadth of avian biodiversity.

## Abstract (web summary)

Lymphatic filariasis and loiasis are diseases caused by insect-borne filarial nematodes. Here, Suh *et al*. identify a retrotransposon that is present in the genomes of these nematodes and seven tropical bird lineages, indicating two waves of horizontal gene transfer around 17–25 million years ago.

## Graphical abstract

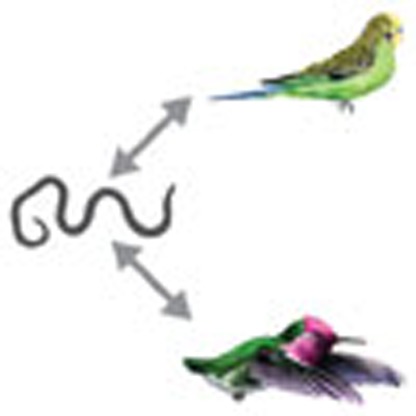

Horizontal transfer (HT) of genetic material has widely shaped eukaryotic genomes^1^ and may often coincide with endosymbiosis^2^ or parasitism^3^. A growing body of evidence suggests that host–parasite relationships are gateways for the HT of transposable elements (TEs), genomic parasites that are unable to leave the cell by their own means. Such exchange of TEs across cellular and organismal boundaries appears to be particularly common in DNA transposons^4^,^5^,^6^, whereas it is rather rare in retrotransposons, which exhibit RNA intermediates^7^,^8^,^9^. The only known case of widespread HT of non-retroviral retrotransposons is that of BovB, a family of retrotransposon-like elements (RTEs) present in the genomes of various mammals and lizards/snakes^10^,^11^, as well as ticks that parasitize them^11^. Further evidence exists for a single HT of CR1 retrotransposons between distant groups of butterflies^12^. However, despite the recent sequencing of many genomes of birds^13^ and filarial nematodes^14^,^15^,^16^,^17^, no evidence of HT exists in the evolutionary history of these species-rich taxa.

Here we report widespread retrotransposon HT between endoparasitic nematodes and the breadth of avian biodiversity in the tropics. Surprisingly, these filarial nematodes are extant endoparasites of humans, and cause lymphatic filariasis and loiasis, which affect ∼170 million people^14^,^16^. We reconstruct the timing and biogeography of these prehistoric host–parasite associations as witnessed by HT events, and thereby propose a novel scenario for the deep origins of two major human diseases.

## Results

### A previously undetected transposon from birds and nematodes

**Figure 1:**
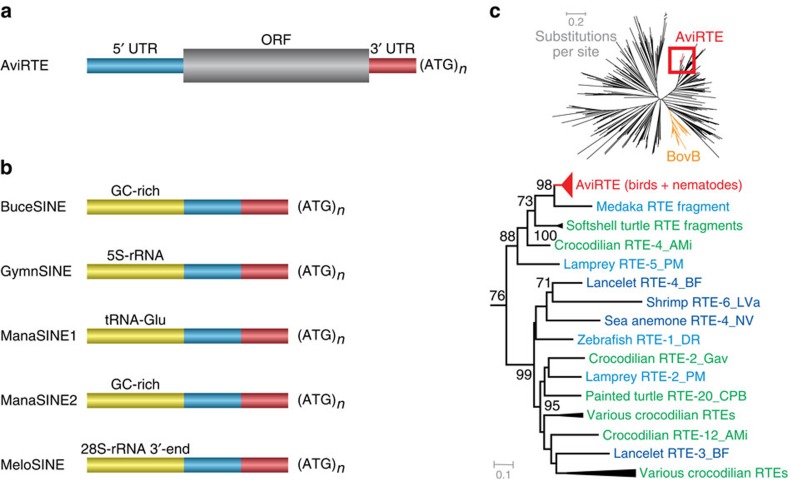
A novel family of RTE retrotransposons from birds and nematodes. (**a**,**b**) Schematic illustration of structural diversity of (**a**) autonomous AviRTE and (**b**) non-autonomous SINEs mobilized by AviRTE. The SINEs consist of a promoter-bearing head (yellow) and a bipartite tail that is derived from part of the 5′ UTR (blue) and the full 3′ UTR (red). Typical RNA polymerase III-transcribed SINEs^21^ were identified within suboscine passerines, where the manakin and tyrant flycatcher lineages share ManaSINE1 with tRNA-Glu gene-derived promoters, whereas the antbird lineage exhibits GymnSINE with 5S-rRNA gene-derived promoters. Furthermore, we detected two emergences of potential SINEs with a GC-rich head including a 5′-GGCCCCGG-3′ motif as a potential protein-binding site^62^; one in the hornbill lineage (BuceSINE) and one in the manakin lineage (ManaSINE2). Another peculiar SINE, MeloSINE, emerged in the budgerigar lineage and exhibits a head derived from the 3′ portion of the 28S-rRNA gene, a configuration similar to a novel SINE recently discovered in mammals^63^. (**c**) Phylogeny (RAxML, GTRCAT model, 1,000 bootstrap replicates, bootstrap values ⩾50% shown) of 370 nucleotide consensus sequences from superfamily RTE (incl. AviRTE and additional BLASTN hits) suggests that AviRTE (red) is distantly related to BovB (orange) and more closely related to RTEs from aquatic animals. RTE subfamilies are in green letters for crocodilians and turtles, in light blue letters for other aquatic vertebrates and in dark blue for aquatic invertebrates. A colour-coded distribution of host taxa across the entire RTE phylogeny is shown in Supplementary Fig. 1.

We describe AviRTE, a novel family of long interspersed elements (LINEs), from bird and nematode genomes. AviRTE belongs to the RTE superfamily, is distantly related to BovB (Fig. 1), and even more distantly related to known nematode RTEs (Supplementary Fig. 1). Instead, AviRTE groups within a diverse set of RTEs from aquatic or semi-aquatic animals (Fig. 1c, Supplementary Fig. 1). Many of these related and recognizable RTE families are from crocodilians and turtles, a pattern that may reflect the low evolutionary rate of these genomes, which are rich in ancient repeats^18^. We initially detected fragments of AviRTE in restriction site-associated DNA (RAD) sequences of a *Zimmerius* flycatcher (Tyrannidae) genome and in BLASTN searches of sequences from other Tyrannidae in GenBank (Supplementary Data 1). These hits are unlikely to be the result of contamination and instead constitute actual TE insertions, because we were able to ascertain orthologous genomic loci of AviRTE presence/absence among multiple species of birds. For example, the ornithine decarboxylase gene exhibits an intronic AviRTE insertion (nested within a 13-bp target site duplication) in some suboscine birds, and an empty insertion site in others (Supplementary Fig. 2). We then examined by BLASTN 48 recently published avian genomes^13^, a wide range of nematode genomes^14^,^15^,^16^,^17^, VectorBase's insect and tick genomes^19^, and GenBank's nucleotide and genome collection (including mammalian genomes). We also survey-sequenced the genomes of three hummingbirds and two additional suboscine passerines. In addition to these screenings, we complemented our taxon sampling by targeted PCR of genomic DNA from various bird species (Supplementary Fig. 3; Supplementary Data 1).

### Horizontal transposon transfer between birds and nematodes

We detected autonomous copies of AviRTE in seven monophyletic clades of birds and two clades of nematodes, but not their respective sister groups (Supplementary Data 1), providing the first evidence for HT in birds and filarial nematodes^20^. Copy numbers range from 141 to 8,306 copies in avian and 273 to 859 copies in nematode genomes (Supplementary Data 1–2). Wherever possible, we reconstructed the respective consensus sequence, revealing a mean overall nucleotide distance of 0.101 substitutions per site between the full-length consensus sequences derived from bird and nematode genomes (Table 1). The high sequence similarity is not restricted to the ∼3.2-kb-long open reading frame, but is also present across the 5′ and 3′ untranslated regions (UTRs) which are ∼800 bp and ∼40 bp in size, respectively. Notably, we also detected evidence for parallel evolution (Supplementary Fig. 4) of non-autonomous, short interspersed elements (SINEs) that are mobilized by the enzymatic machinery of AviRTE LINEs (Fig. 1b, Supplementary Data 1). All these SINEs share a bipartite tail consisting of fragments of the 5′ and 3′ UTRs of AviRTE (Fig. 1b), yet have different promoter-bearing heads (see legend of Fig. 1b). Altogether, the diversity of non-autonomous elements mobilized by AviRTE is the result of lineage-specific SINE emergences (Supplementary Fig. 4) and surpasses the known diversity of SINEs mobilized by the distantly related BovB family^21^.

### The distribution and timing of transposon invasions

**Figure 2:**
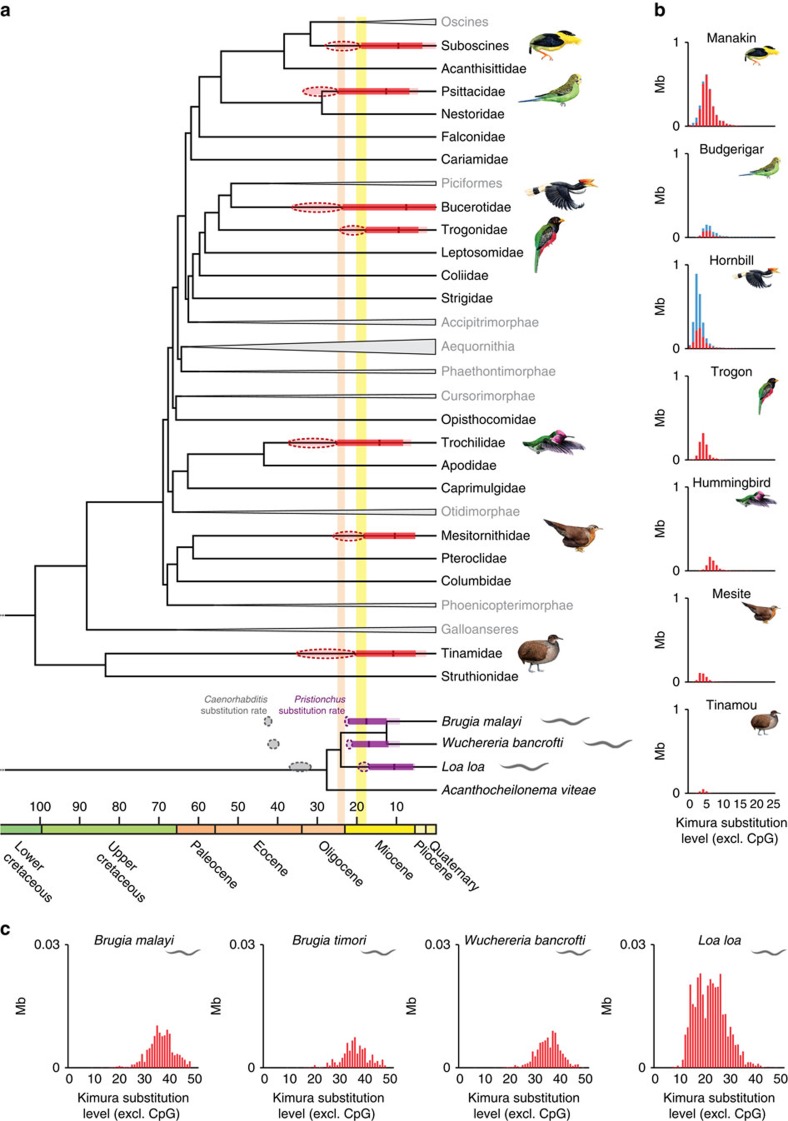
Two waves of horizontal transfer of AviRTE in birds and nematodes. (**a**) Phylogenetic distribution of AviRTE mapped on simplified chronograms of all major avian clades (high-ranking taxon names in grey letters)^22^ and a subtree of filarial nematodes^27^. Together with our dates of AviRTE retrotranspositional activities, this reveals that HT events (red or purple dashed circles denoting minimum and maximum estimates) occurred long after the respective early diversifications of Neoaves and filarial nematodes, putatively in one Oligocene wave (orange) and one Miocene wave (yellow). Dates for AviRTE retrotransposition are either based on a lineage-specific substitution rate of fourfold degenerate sites from the respective bird^13^ (red colour), or a neutral substitution rate from the nematode *Pristionchus pacificus*^23^ (purple colour). For comparison, HT dates based on the mean neutral substitution rate from *Caenorhabditis* spp.^26^ are also shown (grey dashed circles). Minimum estimates for genome invasions and extinctions are the respective start and end points of red or purple lines, respectively, and correspond to the 95% interval of AviRTE retrotranspositional activity measured on the scale of pairwise divergence to consensus (Supplementary Data 2). Also shown are the 99% intervals (light red or light purple lines) as maximum estimates for genome invasions and extinctions, and the mean of activity (tick mark). (**b**,**c**), Landscape plots of AviRTE divergence (red; co-mobilized SINEs in blue) in (**b**) avian and (**c**) nematode genomes illustrate per-genome retrotranspositional activity on a relative time axis. The avian plots are in the same order as the corresponding taxa in the avian phylogeny of panel **a**. We note that all nematode AviRTE plots except the one of *Loa loa* are highly similar in terms of copy numbers (Supplementary Data 1) and mean divergences (Supplementary Data 2), possibly resulting from a single TE invasion of the germline genome of the *Brugia*/*Wuchereria* ancestor.

We then studied the phylogenetic distribution and temporal activity of AviRTE retrotransposition across a dated genome-scale phylogeny of birds^22^. Among the 48 key bird representatives sampled in this phylogeny, AviRTE is present in 7 lineages that span the breadth of avian biodiversity (Fig. 2a). The relatively low sequence divergence between copies (Fig. 2b) and the absence from outgroup genomes of any sequence with even the slightest resemblance to AviRTE (Supplementary Data 1) suggest that this TE family was acquired via HTs long after the Neoaves radiation at the Cretaceous–Paleogene boundary^22^ (Fig. 2a). Given the evidence for very recent AviRTE retrotransposition in some birds (for example, hornbill; Fig. 2b), we reanalysed all genomes for the presence of full-length AviRTE copies and identified zero to six of such elements per genome (Supplementary Data 1). However, all of these copies exhibit multiple frameshifts and premature stop codons (Supplementary Data 3), suggesting that there are no intact ‘master genes' of AviRTE in the sampled genome assemblies.

**Figure 3:**
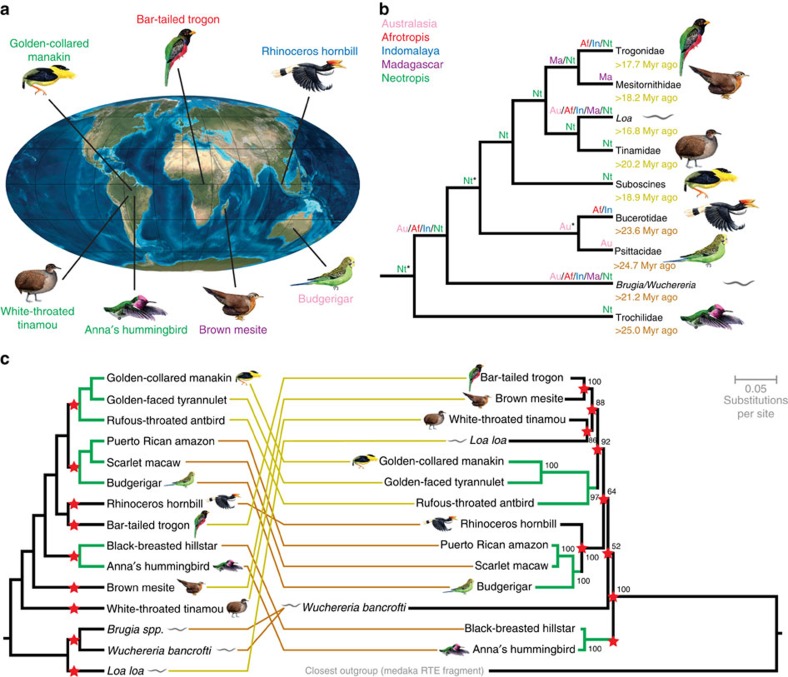
Pantropical transfer of AviRTE putatively mediated by insect-borne nematodes. (**a**) Diverse geographic origins of seven representatives of AviRTE-bearing birds illustrated on a paleogeographic map of the Earth 20 Myr ago. The Mollweide projection map is copyright to Ronald Blakey (used with permission). (**b**) Paleobiogeography of the AviRTE phylogeny (see panel **c**) inferred via statistical dispersal-vicariance (S-DIVA) analysis. We used avifaunal regions *sensu* Ericson^35^. For nodes marked with an asterisk, only those areas which received >33% of the ancestral area distribution on this node are shown. Strikingly, the two waves of HT events (*cf.*, Fig. 2a) are not only separated temporally (genome invasion dates of the first and second waves of HT are shown in orange and yellow letters, respectively), but also phylogenetically and biogeographically. (**c**) The species tree of avian^22^ and nematode^27^ hosts (left) is highly incongruent with the AviRTE phylogeny (right; RAxML, GTRCAT model, 1,000 bootstrap replicates, bootstrap values ⩾50% shown) and the assumption of nine HT events (red asterisks) is required to reconcile the two topologies. The AviRTE phylogeny is rooted to several more or less closely related outgroups (Supplementary Fig. 4; *cf*., Fig. 1c); however, only the closest outgroup is shown for comparability of ingroup internode lengths. Green branches are topologically identical between both trees, implying vertical transmission in species-rich avian lineages. Orange and yellow colours indicate the Oligocene and Miocene waves of HT events, respectively.

We then conservatively inferred minimum times of HTs by applying lineage-specific substitution rates of seven bird species^13^ (derived from dated branch lengths of the corresponding phylogeny^22^) to the upper boundary of the 95% interval of divergences between copies of AviRTE (see Methods). These minimum estimates suggest two temporally distinct bursts of invasions (*t*-test, *P*=0.0006), the first wave >25.0 to >23.6 Myr ago among hummingbirds, psittacid parrots and hornbills (Fig. 2a), and the second wave >20.2 to >17.7 Myr ago in tinamous, suboscine passerines, mesites and trogons. Consistent with the hypothesis of HT involving nematodes, the two waves of HT in birds are temporally compatible with the dates inferred when considering the per-genome divergences of AviRTE copies in nematode genomes (Fig. 2c) under a neutral substitution rate^23^ and a generation time of 90 days^24^. Accordingly, genome invasions are inferred to have taken place >21.2 Myr ago in the ancestor of *Brugia* spp./*Wuchereria bancrofti*, the causative agents for lymphatic filariasis, and >16.8 Myr ago in the ancestor of *Loa loa*, the causative agent for loiasis. In contrast to the aforementioned lineage-specific substitution rates of birds, the nematode HT dates are based on the neutral substitution rate of a mutation accumulation line from a different nematode, *Pristionchus pacificus*^23^, because such rates are unavailable for filarial nematodes. Although Weller *et al*.^23^ suggested the rate in *Pristionchus* to be representative for nematodes, we emphasize that molecular dating of nematodes is notoriously difficult due to differences in life style and a virtually non-existent fossil record^25^, and our nematode dates of AviRTE transfers should therefore be treated with caution. Nevertheless, these nematode HT dates, and dates derived from the slightly lower neutral substitution rates of *Caenorhabditis* species^26^ both suggest temporally distinct invasions in the *Brugia*/*Wuchereria* lineage and the *Loa* lineage (Fig. 2a, Supplementary Data 2). We also note that the *Pristionchus*-based minimum dates are temporally compatible with the split of the *Brugia*/*Wuchereria*/*Loa* lineage from *Acanthocheilonema viteae* and other AviRTE-free outgroups (Fig. 2a, Supplementary Data 1) in an independently dated nematode phylogeny^27^. Furthermore, grouping the HT dates from the *Brugia*/*Wuchereria* lineage in the first and *L. loa* in the second of the aforementioned waves of HT again suggests that AviRTE transfers occurred in two distinct bursts (*t*-test, *P*=0.0002). The two independent genome invasions of nematodes are surprising given that these two lineages are closely related within filarial nematodes^27^,^28^, but in agreement with differences between the shapes of the AviRTE divergence landscapes of the *Loa* lineage and the *Brugia*/*Wuchereria* lineage (Fig. 2c, Supplementary Data 1–2). Finally, the very short internodes in the AviRTE phylogeny indicate a rapid succession of HTs in birds and nematodes (Fig. 3c), and there is phylogenetic evidence that these two bursts of HT are discrete. More precisely, AviRTE subfamilies of the second wave form a monophyletic group nested within the first wave, and the nematode AviRTEs group with avian AviRTEs of similar minimum invasion dates (Fig. 3b).

### The paleobiogeography of transposon invasions

Although the AviRTE-bearing lineages of birds span the entirety of the avian Tree of Life, it is striking that all of these mainly occur in tropical regions^29^. They include typical Neotropical avifauna such as hummingbirds and tinamous, the Madagascan endemic mesites and members of more widespread tropical bird assemblages (Fig. 3a,b). It was recently noted that genome-scale dating of birds yields much lower divergence estimates than other molecular studies^22^,^30^,^31^, which explains why our inferred avian HT dates postdate, for example, previous dates for the diversifications of suboscine passerines^32^ and psittacid parrots^33^. However, irrespective of this discrepancy between absolute dates, our dense taxon sampling suggests that HTs occurred in the respective ancestors of Psittacidae (psittacid parrots), Suboscines (suboscine passerines) and Trochilidae (hummingbirds); and potentially Bucerotidae (hornbills), Mesitornithidae (mesites), Tinamidae (tinamous) and Trogonidae (trogons) (Supplementary Data 1, Fig. 3c). These relative dates predate the onsets of lineage-specific diversification and thus permit paleobiogeographic inferences. We reconstructed the ancestral areas of HTs by assuming a pantropical distribution of filarial nematodes and considering existing evidence for the paleobiogeography of the respective avian clades. Passerines and parrots are more closely related than previously thought^22^,^34^ and diversified in Australasia^33^,^35^ (but see ref. 30). However, suboscine passerines likely originated in the Neotropics^30^,^32^, while the biogeographic origins of hornbills and trogons are unknown^35^. We find a Neotropical origin of AviRTE is likely given the deep branching of hummingbirds in the AviRTE phylogeny (Fig. 3b), and that the remainder of the first wave of HT (Fig. 2a) occurred across all tropical regions except Madagascar (see Methods and Supplementary Fig. 5 for geographically less constrained analyses). In contrast, the second wave of HT (Fig. 2a) includes Madagascar and took place predominantly in the Neotropics. Altogether, these results suggest that the paleobiogeography of AviRTE transfers occurred on a global, pantropical scale. It is further worth noting that the respective phylogenetic positions of AviRTEs from *Brugia* spp./*W. bancrofti* and *L. loa* coincide with the aforementioned temporal similarities in genome invasion dates. We therefore propose that the ancestor of *Brugia*/*Wuchereria* was involved in the first burst of AviRTE transfers, while the *Loa* ancestor was part of the second wave of HT. In addition, direct comparison of the host species tree with the AviRTE tree (Fig. 3c) suggests that the differences in phylogenetic topology are most parsimoniously explained by nine HT events. However, the TE relationships within hummingbirds, psittacid parrots and suboscine passerines appear to be the result of vertical inheritance, respectively, suggesting that AviRTE shaped the genome evolution of these species-rich lineages by persistence of retrotranspositional activity across their diversifications (Supplementary Data 2).

### Possible vectors for HT

This study is the first to report HT of TEs involving the genomes of birds or filarial nematodes. Our results provide phylogenetic, paleobiogeographic and temporal evidence that the endoparasitic *Brugia*/*Wuchereria* and *Loa* lineages each were involved in AviRTE transfer. These nematodes presently have a near-pantropical distribution and are transmitted by mosquitoes^14^,^27^ and deerflies^16^, respectively. Although most birds are capable of flight, their dispersal has been historically rather limited across avifaunal boundaries, especially in the tropics^29^,^35^. We therefore suggest that the two bursts of pantropical exchanges of AviRTE between five avifaunal regions were catalysed by the pantropical dispersal potential of filarial nematodes via their dipteran vectors. While it is conceivable that AviRTE transfer occurred directly from blood-sucking dipterans to birds, we find that AviRTE is absent from available dipteran genomes sequences^19^ and endoparasitic interaction between insect-borne nematodes and birds may be a more plausible platform for such rampant HT. Irrespective of the macroscopic vectors for HT, however, it remains mysterious as to how exactly TEs move from one germline genome into another, with potential candidates being naked RNA or viruses^8^. Alternatively, intracellular *Wolbachia* bacteria are plausible cell-penetrating vectors^8^ and infect many filarial nematodes, yet they are absent in *L. loa*^16^. It is further worth noting that successful HT is much more complex than the mere infiltration of a new host cell by transposon DNA or retrotransposon RNA. For ancestrally transferred AviRTEs to be visible in extant genomes, a full-length retrotransposon RNA has to colonize a new germline genome, retrotranspose into a genomic environment which permits retrotranspositional activity as an intact AviRTE ‘master gene' and drift to fixation in the host population. It is therefore likely that the nine events of successful HT and germline infiltration, reconstructed here from dozens of sampled animal genomes, is but a small fraction of the actual number of prehistoric AviRTE exchanges between birds and filarial nematodes.

## Discussion

Our study supports the notion that host–endoparasite interactions are prone to episodic gene exchange, including ‘selfish genes' such as AviRTE and other TEs, a process that passes on genetic material as ‘public goods'^36^ among unrelated organisms. We demonstrate that HTs bear witness of long-extinct organismal interactions between birds and nematodes, although it remains undetermined whether the interactions leading to bird–nematode HTs were direct or indirect. The causative agents of lymphatic filariasis and loiasis infect humans as their adult host^14^,^16^ and many other filarial nematodes are known to only infect mammals, possibly the result of an ancestral mammalian host range^27^. The absence of AviRTE in mammalian genomes despite extensive bird–nematode HT seems to challenge this view. The two waves of HT spanned the avian Tree of Life and involved rapid movement among all five avifaunal regions of the tropics, which is puzzling given that tropical landbirds have limited propensities for inter-continental dispersal^35^. We thus hypothesize that the *Brugia*/*Wuchereria* and *Loa* lineages were Oligocene/Miocene parasites of tropical birds and dispersed pantropically through their dipteran vectors. This may explain the aforementioned complex paleobiogeography of HTs. Such a scenario requires that the nematode lineages underwent two subsequent host switches to humans or their hominid ancestors, likely after AviRTE ceased retrotranspositional activity in filarial nematodes and thus lost its potential for HT into hominid genomes. Our indirect evidence for ancient interactions between birds and the ancestors of the causative agents of lymphatic filariasis and loiasis raises the possibility that these widespread human pathogens may have independently evolved from prehistorically ubiquitous bird endoparasites. We anticipate that exploring the neglected biodiversity of extant bird-infecting nematodes will add further support to this hypothesis.

## Methods

### *In silico* screening

We initially detected a putatively RTE-mobilized SINE (later termed ‘ManaSINE1') in RAD sequences of a Neotropical *Zimmerius* flycatcher^37^. The SINE sequence was BLASTN^38^ searched against a budgerigar^39^ repeat library that we had generated *de novo* using Repeatmodeler version 1.0.5 (http://www.repeatmasker.org/RepeatModeler.html). This led to the discovery of a nearly full-length consensus sequence of an autonomous RTE which we termed ‘AviRTE'. We then used the budgerigar AviRTE fragment as query for all subsequent BLASTN screens (cutoff *e*-value 1e–10) of animal genome assemblies and nucleotide sequences available in GenBank^40^, including the genomes of 48 birds^13^ and 2 additional parrots^41^,^42^. Furthermore, our screenings comprised all insect and tick genomes in VectorBase^19^, and all filarial nematode genomes in WormBase^15^,^17^. We made sure that, for each of the bird and nematode clades exhibiting AviRTE, we also sampled the closest relatives as outgroups (Supplementary Data 1).

### *In vitro* screening

Our taxon sampling was complemented by species where genome or survey sequences were unavailable (Supplementary Data 1). We sampled these using a short PCR that amplifies a 126-bp region from the conserved 5′ UTR of AviRTE. PCR parameters were 40 cycles of 94 °C for 20 s, 53 °C for 45 s and 68 °C for 60 s, followed by final elongation for 120 s at 68 °C. We used the primers AviRTEint-F/R (5′- CCTGAGGACTTCACTGTCACC -3′+5′- CTTCAAGCCTGTGCAGTGG -3′) and interpreted the absence of an amplicon as an indication of genomic absence of AviRTE (Supplementary Fig. 3). In the case of *Pitta moluccensis*, we additionally confirmed the AviRTE presence by direct Sanger sequencing of the PCR amplicon (Supplementary Data 4). Finally, we were able to amplify the full length of AviRTE in *Gymnopithys rufigula* with four overlapping PCR amplicons and subsequent Sanger sequencing of four clones per amplicon, permitting the generation of a consensus sequence. PCR parameters were an initial denaturation for 120 s at 94 °C, 35 cycles of 94 °C for 30 s, 50/54 °C for 30 s and 72 °C for 80 s, followed by final elongation for 300 s at 72 °C. Purified PCR products were cloned into *Escherichia coli* JM09 cells using the pGEM-T Vector, followed by PCR amplification via standard M13 primers. The four primer pairs were AviRTEfull-1F/R (5′- TCGTGGGGAAAGAGCTTG -3′+5′- AATACAATCGGAATGACCTGTC -3′), AviRTEfull-2F/R (5′- AGGCATCTCTCAGGAGTTGG -3′+5′- CATAGAATCCTCTGTGGTCACC -3′), AviRTEfull-3F/R (5′- CAAGTGGTGGATCAACCTAGC -3′+5′- TGATTTAGGGTCTTGGTGTGG -3′), and AviRTEfull-4F/R (5′- CCTATTCAATCTAAGGCGACTG -3′+5′- ATCATCATGGCTTGGCTTC -3′).

### Whole-genome survey sequencing

We obtained whole-genome survey sequences via paired-end sequencing on the Illumina HiSeq 2000 platform (100-bp reads). The insert size was 300 bp and the final coverage ∼6 × for the two suboscines. For the three hummingbirds, the insert sizes ranged from 275 to 450 bp and the final coverage was ∼0.1 × . Genomic presence of AviRTE was ascertained by BLASTN screens (cutoff *e*-value 1e–10). In the case of *Oreotrochilus melanogaster* and *Zimmerius chrysops*, we were able to infer complete and near-complete AviRTE consensus sequences, respectively.

### Consensus sequences

Majority-rule consensus sequences were generated manually from each AviRTE-bearing host genome assembly. We used standard procedures^43^,^44^ to reconstruct full-length AviRTEs via BLASTN and extension by re-BLASTN searches to overcome incomplete 5′ and 3′ ends. For each of these multiple rounds of BLASTN searches, multiple sequence alignments of BLASTN hits were constructed using MAFFT^45^,^46^ version 7 (E-INS-i, http://mafft.cbrc.jp/alignment/server/index.html). The resultant consensus sequences span the full length of AviRTE or co-mobilized SINEs, in rare cases with ‘N' nucleotides in ambiguous regions that resisted reconstruction. Full-length consensus sequences of AviRTEs and co-mobilized SINEs were submitted to Repbase (http://www.girinst.org/repbase/index.html) (see also Supplementary Data 5).

### Phylogenetic analyses

We automatically aligned consensus sequences using MAFFT and then manually realigned ambiguous regions. For the AviRTE phylogenies (Fig. 3c, Supplementary Fig. 4), this was sufficient for generating nucleotide sequence alignments across the full length of AviRTE (Supplementary Data 6). On the other hand, the nucleotide sequence alignment for the phylogeny of all RTE subfamilies present in RepBase, additional GenBank BLASTn hits and all AviRTE subfamilies (Fig. 1c, Supplementary Fig. 1) contained many ambiguities and poorly aligned regions that were removed using Gblocks^47^ version 0.91b. We chose standard parameters in the Gblocks webserver (http://molevol.cmima.csic.es/castresana/Gblocks_server.html) for less stringent selection of alignment positions (that is, smaller final blocks, gap positions within the final blocks, less strict flanking positions), yielding a 429-bp high-confidence alignment (Supplementary Data 7) from the original 23,637 bp. Note that the low number of retained alignment positions reflects the fact that very distant RTE subfamilies were included, such as those from angiosperm plants. We excluded sequences that aligned poorly at the nucleotide level or comprised <200 bp of the filtered alignment, reducing the total amount of sequences in the RTE superfamily alignment from 444 to 370. We then conducted all phylogenetic analyses under maximum likelihood in RAxML^48^ version 8.1.11 (GTRCAT model, 1,000 bootstrap inferences) on the CIPRES Science Gateway^49^ (https://www.phylo.org/portal2/login!input.action). All phylogenetic trees are available in Newick format (Supplementary Data 8).

### Distance and dating analyses

Pairwise nucleotide distances between AviRTE consensus sequences (Table 1) were calculated in MEGA6 (ref. 50) under the Kimura 2-parameter model^51^ with uniform rates among sites and pairwise deletion of gaps/missing data.

We then annotated the genomic copies of AviRTE using RepeatMasker version 3.3.0 (http://www.repeatmasker.org/RMDownload.html) with a custom repeat library for each of the AviRTE-bearing genome assemblies. This library contained the conspecific AviRTE consensus (and, if present, co-mobilized SINE consensus sequences). In the cases where this sequence was incomplete and contained ‘N' residues, we instead used the full-length consensus sequence from the most closely related host genome. We subsequently calculated per-copy distances to consensus in the calcDivergenceFromAlign.pl script included in the RepeatMasker program package (Kimura 2-parameter model, excluding CpG sites) and plotted these as AviRTE divergence landscapes (Fig. 2b,c). Such a divergence distribution reflects the retrotranspositional activity of AviRTE on a relative time scale per genome. However, we hypothesize that its high-divergence and low-divergence extrema, which usually comprise merely <100 bp per divergence bin (Supplementary Data 2), may in fact arise from genomic outliers in substitution rates, such as conserved or hypervariable regions. In addition, it is plausible that some of the very short, high-divergence AviRTE fragments result from spurious hits to random non-AviRTE sequence during the BLAST-based RepeatMasker annotation. We therefore considered the boundaries of 95% of the distribution as suitable conservative estimates for the onset (latest point of genome invasion) and end (earliest point of extinction) of AviRTE retrotransposition, and the 99% interval as the maximum duration (Supplementary Data 2). Absolute dates were inferred by dividing these divergence values by two times the substitution rate (see Supplementary Data 2 for more details). For the seven birds, we used the fourfold degenerate site substitution rate derived from the respective bird genome^13^,^52^. Given the lack of substitution rates from filarial nematodes, we considered the neutral substitution rates of different nematodes, *P. pacificus*^23^ and *Caenorhabditis* species^26^, under the assumption of a generation time of 90 days for filarial nematodes^24^. Finally, we plotted the inferred AviRTE retrotranspositional activities on the dated genome-scale tree from the study by Jarvis *et al*.^22^,^53^ (Fig. 2a).

### Biogeographic analyses

We used the S-DIVA method^54^ for biogeographic reconstruction of the site of HT occurrence under the assumption that extant bird species usually occur in less than two avifaunal regions^35^. Thus, analyses were done in RASP^55^ using standard parameters and allowing a maximum of two areas per node. Given the current cosmopolitan distribution of nematodes and their great age compared with birds^56^, we assumed a pantropical distribution of the *Loa* and *Brugia*/*Wuchereria* lineages at the time of HT. For birds, we considered existing evidence for the paleobiogeography of the respective avian clades to infer the areas each lineage inhabited at the time of the HT of AviRTE. Because there is no certainty about the paleobiogeography of each avian lineage, we carried out three different analyses at various levels of conservatism to infer the main location of HT.

First, we considered the widest possible distribution of each bird lineage at the time of HT (Supplementary Fig. 5a). In the absence of a detailed avian fossil record^57^, this meant that the area of occurrence of most bird lineages would be equated to their present-day distribution, such as hummingbirds (Trochilidae) in the Neotropics; psittacid parrots (Psittacidae) in Australasia, Africa, Indomalaya and the Neotropics; tinamous (Tinamidae) in the Neotropics; mesites (Mesitornithidae) in Madagascar; and trogons (Trogonidae) in Africa, Indomalaya and the Neotropics. We made an exception for the paleotropical hornbills (Bucerotidae) and the pantropical suboscine passerines (Suboscines): both lineages occur in Australasia, but do so only marginally with one and five species, respectively, all of which are known to be of fairly recent Indomalayan descent^58^,^59^. Therefore, hornbills were only coded for Africa and Indomalaya, whereas suboscines were only coded for Africa, Indomalaya and the Neotropics in this analysis.

Second, we repeated the first analysis but additionally included areas in which lineages may no longer be present now but are thought to have occurred around the time of HT based on the fossil record (Supplementary Fig. 5b). Given the poor avian fossil record^57^, this only changed the area designation for a single lineage, the hummingbirds, which are presently distributed only in the New World^60^ but are known from early-Oligocene Old World fossils^61^. Hence, in this analysis we coded hummingbirds for the Neotropics, Africa and Indomalaya.

Finally, in our main analysis (Fig. 3b) we only included regions thought to be the areas of occurrence of each respective bird clade roughly at the time of HT based on the current literature. For lineages in which the area of occurrence at the time of HT could not be further narrowed down in comparison to present-day distribution (that is trogons, mesites, tinamous, hornbills and hummingbirds), the same areas as in the first analysis (Supplementary Fig. 5a) were used. However, for psittacid parrots (Psittacidae), we used Australasia as the area of occurrence based on two considerations: (i) there is ample phylogenetic evidence on the Australasian origin of most deep parrot lineages^33^,^35^; and (ii) the internal topology of psittacid AviRTE subfamilies strongly suggests that the HT occurred into the last common ancestor of Psittacidae before the divergence of this family into Neotropical, African and Australasian clades. In the same way, we used the Neotropics as the area of occurrence for suboscines based on two similar considerations: (i) the Neotropics are the most likely ancestral area of suboscines^35^,^58^; and (ii) the internal topology of suboscine AviRTE subfamilies, as well as their presence in both major suboscine clades strongly suggest that the HT occurred into their last common ancestor before the break-up into Old World and New World suboscines.

## Additional information

**Accession codes:** Raw survey sequence data have been deposited in the Sequence Read Archive (SRA) with accession codes SRS1259545 to SRS1259546, and SRS1303252 to SRS1303254.

**How to cite this article:** Suh, A. *et al*. Ancient horizontal transfers of retrotransposons between birds and ancestors of human pathogenic nematodes. *Nat. Commun.* 7:11396 doi: 10.1038/ncomms11396 (2016).

## Supplementary Material

Supplementary Information Supplementary Figures 1-5 and Supplementary References.

Supplementary Data 1 Taxon sampling of birds and nematodes used for the detection of AviRTE.

Supplementary Data 2 Activity periods of AviRTE derived from per-copy distances in bird and nematode genomes.

Supplementary Data 3 Amino acid alignment of AviRTE ORF from subfamily consensus sequences and single full-length copies. In addition to premature stop codons, all of the single full-length copies contain frameshifts, which was compensated by the insertion of alignment gaps prior to ORF translation.

Supplementary Data 4 Alignment of the AviRTE 5' UTR region amplified by short PCR. Primer sequences and the Old World suboscine Pitta moluccensis are included.

Supplementary Data 5 Fasta-formatted consensus sequences of AviRTE subfamilies and co-mobilized SINEs.

Supplementary Data 6 Nucleotide sequence alignment of AviRTE subfamilies, co-mobilized SINEs, and selected RTE outgroups. This alignment was used for phylogenetic analyses shown in Supplementary Fig. 4 and, after exclusion of SINEs, in Fig. 3c.

Supplementary Data 7 Nucleotide sequence alignment of AviRTE subfamilies, all RTE families present in RepBase, and additional GenBank BLASTn hits. This alignment was used for the phylogenetic analysis shown in Fig. 1c and Supplementary Fig. 1.

Supplementary Data 8 Newick-formatted phylogenetic trees from (a) Fig. 1c and Supplementary Fig. 1, (b) Fig. 3c, and (c) Supplementary Fig. 4.

## Figures and Tables

**Table 1:** Pairwise distances between full-length AviRTE consensus sequences.

**AviRTE consensus from host**	**apaVit**	**araMac**	**bucRhi**	**calAnn**	**gymRuf**	**loaLoa**	**manVit**	**melUnd**	**mesUni**	**oreMel**	**tinGut**
*Apaloderma vittatum*											
*Aca macao*	0.087										
*Buceros rhinoceros*	0.078	0.055									
*Calypte anna*	0.080	0.099	0.089								
*Gymnopithys rufigula*	0.090	0.112	0.105	0.107							
*Loa loa*	0.109	0.135	0.131	0.134	0.146						
*Manacus vitellinus*	0.116	0.139	0.131	0.138	0.133	0.169					
*Melopsittacus undulatus*	0.101	0.065	0.074	0.116	0.132	0.152	0.151				
*Mesitornis unicolor*	0.035	0.091	0.084	0.084	0.090	0.114	0.118	0.108			
*Oreotrochilus melanogaster*	0.076	0.094	0.086	0.009	0.104	0.129	0.135	0.114	0.081		
*Tinamus guttatus*	0.047	0.086	0.077	0.081	0.088	0.103	0.117	0.102	0.051	0.078	

Note that only full-length consensus sequences without 'N' residues were included in this analysis.
