# Supplementary material for: Ancient horizontal transfers of retrotransposons between birds and ancestors of human pathogenic nematodes
Source: Nat Commun. 2016 Apr 21;7:11396. doi: 10.1038/ncomms11396 (PMC4844689; doi:10.1038/ncomms11396)
Supplement: Supplementary Information — Supplementary Figures 1-5 and Supplementary References. [file ncomms11396-s9.pdf]

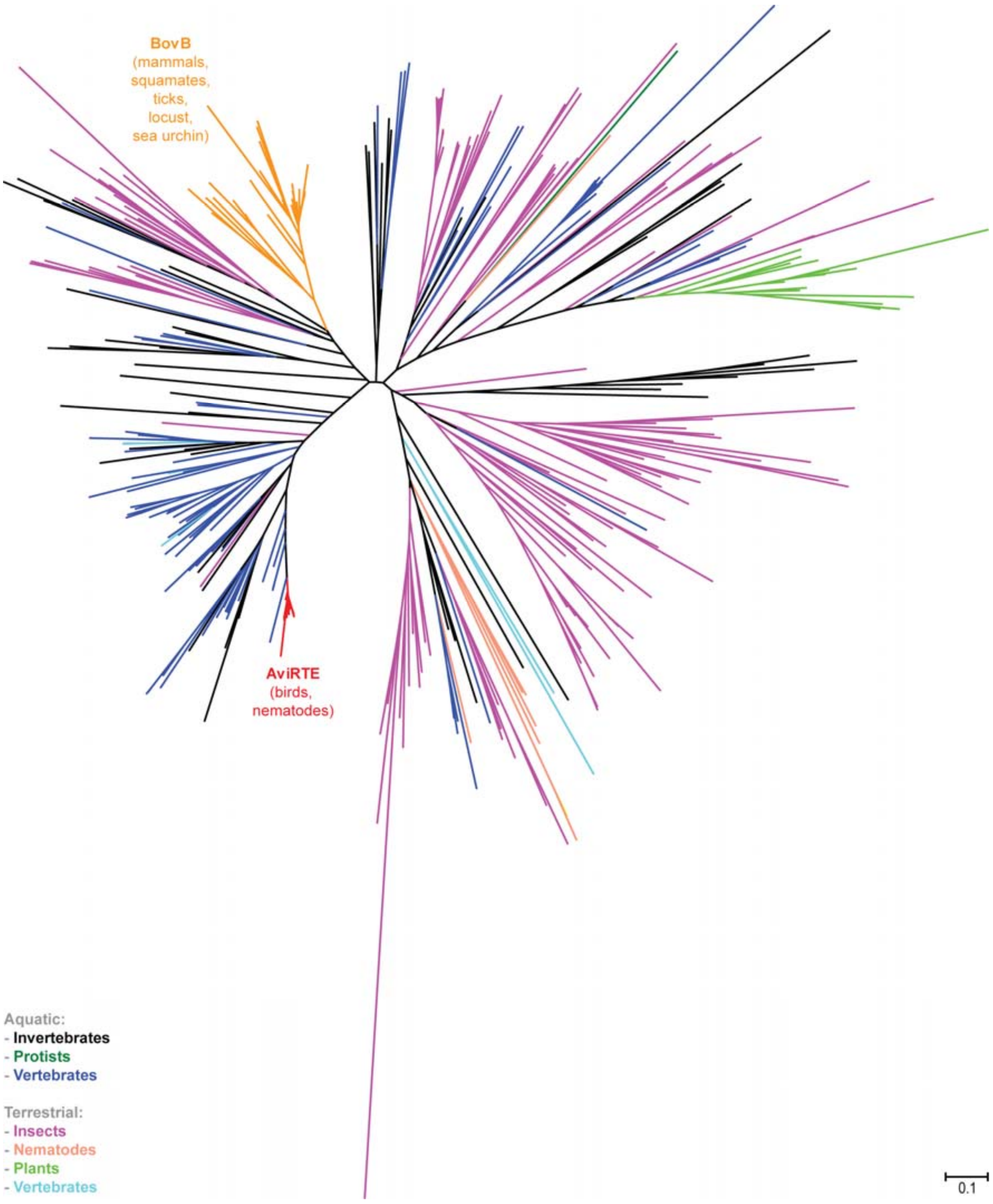

Supplementary Figure 1 | Detailed phylogenetic position of AviRTE within the RTE phylogeny of Figure 1c.

AviRTE (red) is distantly related to BovB (orange) and known nematode RTEs (pink). The RTE phylogeny (GTRCAT model, 1,000 bootstrap replicates, bootstrap values ≥50% shown) is based on a nucleotide sequence alignment of 370 RTE subfamilies after excluding poorly aligned positions and sequences.

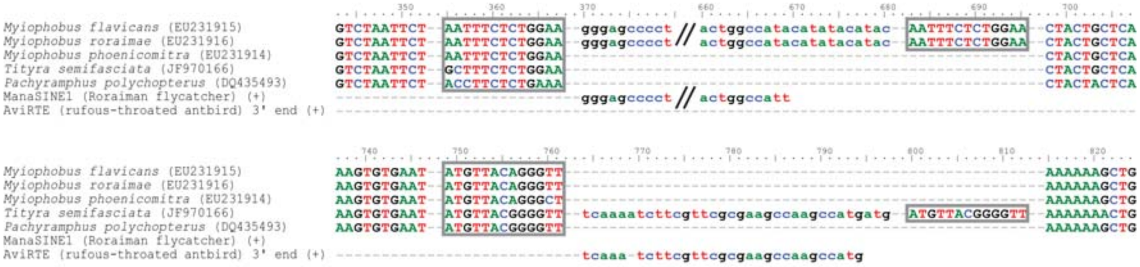

Supplementary Figure 2 | Presence/absence alignment of AviRTE and ManaSINE1 insertions in suboscine passerines.

Shown is a partial alignment of the ornithine decarboxylase gene (exons 6 through 8) with a selection of sequences from New World flycatchers available in GenBank. TE-derived nucleotides are shown in lowercase letters and flanked by 13-nt target site duplications (boxed). High sequence similarity to consensus and perfect target site duplications suggest very recent retrotransposition of AviRTE and ManaSINE1 in New World flycatchers. We anticipate their utility as presence/absence markers for phylogenomics[1] and population genomics[2] of birds.

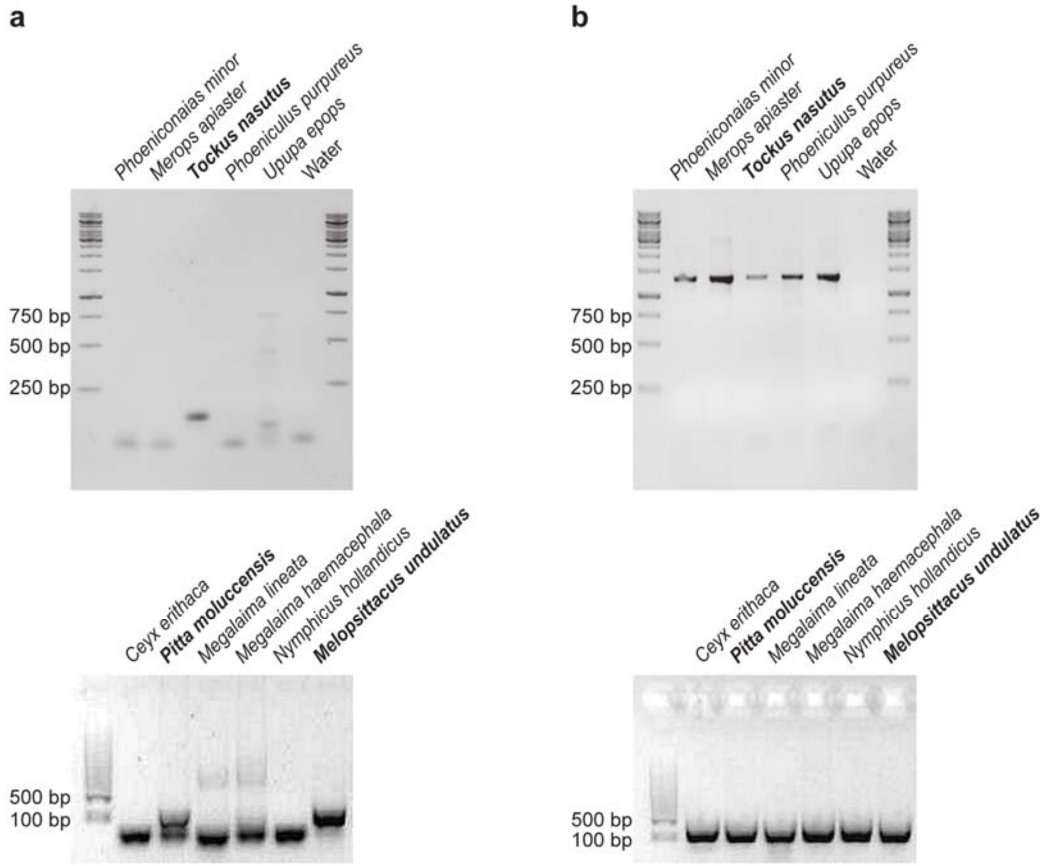

Supplementary Figure 3 | AviRTE detection via short PCR.

a, Detection of genomic presence of AviRTE (bold sample names) via amplification of a 126-bp region from the conserved 5’ UTR of AviRTE. The primers were AviRTEint-F (5’- CCTGAGGACTTCACTGTCACC-3’) and AviRTEint-R (5’-CTTCAAGCCTGTGCAGTGG-3’). **b**, Control reactions amplifying a fragment of the mitochondrial *cytochrome c oxidase I* gene (*cox1*). We used the primers L7987 (5'-TCHGAYTAYCCWGAYGCNTAYAC-3') + H9235 (5'-TCRAAGAAGCTTAGGTTCATGGTCA-3') (http://people.bu.edu/msoren/Bird.mt.Primers.pdf) in the upper panel, and AWC F1 (5'-CGCYTWAACAYTCYGCCATCTTACC-3') + AWC intR1 (5'-CCTGGTTGACCTAGTTCTGCTCG-3')[3] in the lower panel. The inverted photos were taken after gel electrophoresis with 1% agarose gels.

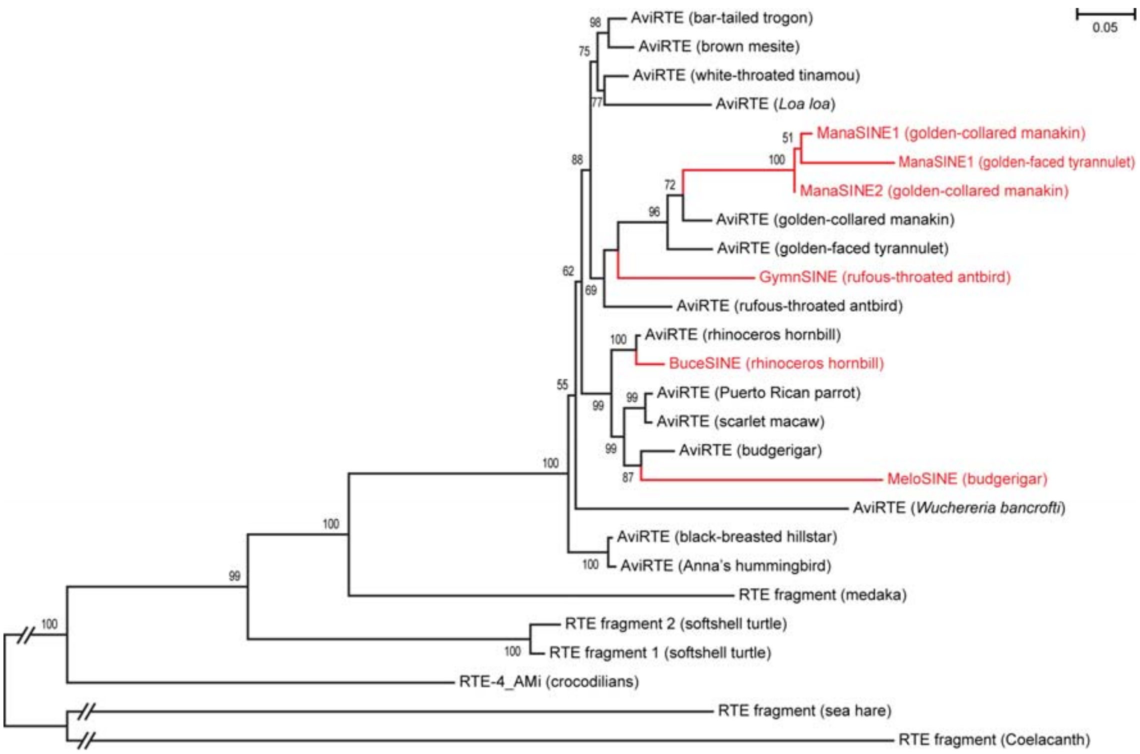

Supplementary Figure 4 | Phylogenetic evidence for parallel evolution of AviRTE-mobilized SINEs.

The AviRTE phylogeny (RAxML, GTRCAT model, 1,000 bootstrap replicates, bootstrap values ≥50% shown) contains the five identified SINEs mobilized by AviRTE and is topologically identical to the phylogeny of autonomous AviRTE subfamilies only (Fig. 3b).

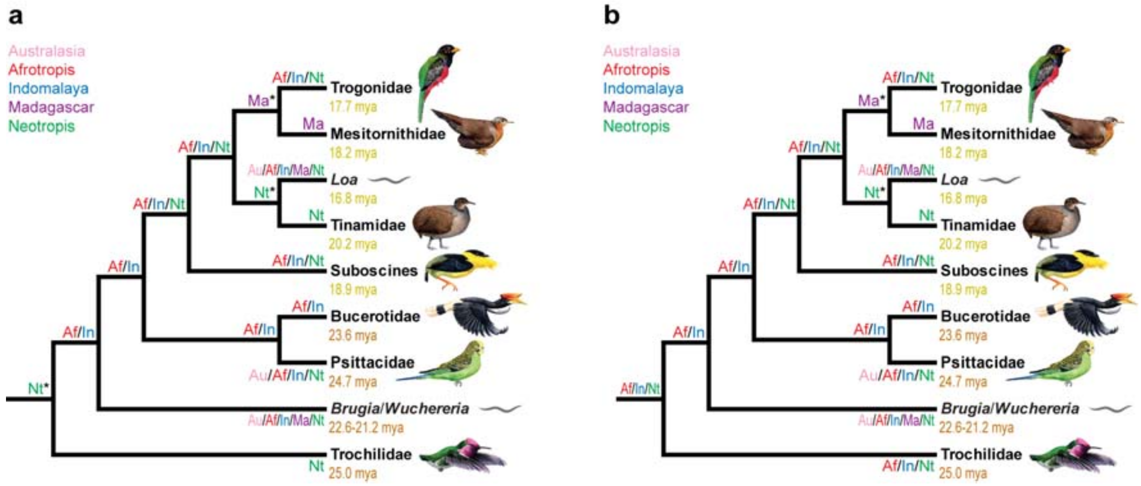

Supplementary Figure 5 | Paleobiogeography of AviRTE using less constrained ancestral areas.

a, S-DIVA analysis with maximal ancestral areas for suboscine passerines and psittacid parrots based on their main present-day distributions[4,5]. For nodes marked with an asterisk, only those areas are shown which received >33% of the ancestral area distribution on this node. **b**, S-DIVA analysis with maximal ancestral areas for suboscine passerines, psittacid parrots, and hummingbirds. Although the crown group of hummingbirds is clearly of Neotropical origin[6], fossils document the presence of stem-group hummingbirds in the Old World during the early Oligocene[7]. Genome invasion dates of the first and second bursts of HT are shown in orange and yellow letters, respectively.

Supplementary References

1 Suh, A. *et al.* Mesozoic retroposons reveal parrots as the closest living relatives of passerine birds. *Nat. Commun.* **2**, 443 (2011).

2 Suh, A., Kriegs, J. O., Donnellan, S., Brosius, J. & Schmitz, J. A universal method for the study of CR1 retroposons in nonmodel bird genomes. *Mol. Biol. Evol.* **29**, 2899–2903 (2012).

3 Patel, S., Waugh, J., Millar, C. D. & Lambert, D. M. Conserved primers for DNA barcoding historical and modern samples from New Zealand and Antarctic birds. *Mol. Ecol. Resour.* **10**, 431–438 (2010).

4 Ericson, P. G. *et al.* A Gondwanan origin of passerine birds supported by DNA sequences of the endemic New Zealand wrens. *Proc. R. Soc. B* **269**, 235–241 (2002).

5 Schweizer, M., Seehausen, O. & Hertwig, S. T. Macroevolutionary patterns in the diversification of parrots: effects of climate change, geological events and key innovations. *J. Biogeogr.* **38**, 2176–2194 (2011).

6 McGuire, J. A. *et al.* Molecular phylogenetics and the diversification of hummingbirds. *Curr. Biol.* **24**, 910–916 (2014).

7 Mayr, G. Old World fossil record of modern-type hummingbirds. *Science* **304**, 861–864 (2004).
